# Tenecteplase real-world data: A three phase sequential comparison

**DOI:** 10.1177/23969873231187436

**Published:** 2023-07-25

**Authors:** Anna Ranta, Alicia Tyson, Bhavesh Lallu, Teddy Y Wu, Martin Punter, Csilla Manoczki, John Chalissery, Akesh Pillai, Karim Mahawish, Roldan Conde, Marianne Falconer, Karyn Wills, Chaminda Gunawardana, Suzanne Busch, John Gommans

**Affiliations:** 1 Wellington Regional Hospital, Wellington, New Zealand; 2 University of Otago, Wellington, New Zealand; 3 Taranaki Base Hospital, New Plymouth, New Zealand; 4 Christchurch Hospital, Christchurch, New Zealand; 5 Whanganui Hospital, Whangarei, New Zealand; 6 Palmerston North Hospital, Palmerston North, New Zealand; 7 Nelson Hospital, Nelson, New Zealand; 8 Hutt Valley Hospital, Lower Hutt, New Zealand; 9 Hawke’s Bay Hospital, Hastings, New Zealand

**Keywords:** Stroke, reperfusion, thrombolysis, tenecteplase

## Abstract

**Introduction:**

The New Zealand (NZ) Central Region Stroke Network, serving 1.17 million catchment population, changed to tenecteplase for stroke thrombolysis in 2020 but was forced to revert to Alteplase in 2021 due to a sudden cessation of drug supply. We used this unique opportunity to assess for potential before and after temporal trend confounding.

**Patients and methods:**

In NZ all reperfused patients are entered prospectively into a national database for safety monitoring. We assessed Central Region patient outcomes and treatment metrics over three time periods: alteplase use (January 2018–January 2020); during switch to tenecteplase (February 2020–February 2021) and after reverting to alteplase (February 2021–December 2022) adjusting regression analyses for hospital, age, onset-to-needle, NIHSS, pre-morbid mRS and thrombectomy.

**Results:**

Between January 2018 and December 2022, we treated 1121 patients with Alteplase and 286 with tenecteplase. Overall, patients treated with tenecteplase had greater odds of favorable outcome ordinal mRS [aOR = 1.43 (95% CI = 1.11–1.85)]; shorter door-to-needle (DTN) time [median 52 (IQR 47–83) vs 61 (45–84) minutes, *p* < 0.0001] and needle to groin (NTG) times [118 (74.5–218.5) vs 185 (118–255); *p* = 0.02)]. Symptomatic intracerebral hemorrhage (sICH) rate was lower in tenecteplase group [aOR 0.29 (0.09–0.95)]. Findings similarly favored tenecteplase when comparing tenecteplase to only the second alteplase phase. There was no inter-group difference when comparing the two alteplase phases.

**Conclusions:**

Our results suggest that previously reported benefits from tenecteplase in a real-world setting were not likely attributable to a temporal confounding.

## Introduction

Increasing clinical trial and real-world data supports the use of tenecteplase as a viable alternative to alteplase for stroke thrombolysis.^1–5^ Due to tenecteplase’s practical advantages of a single bolus injection, non-inferiority alone is a compelling reason to make the change although some evidence suggests TNK might be superior especially in patients with large vessel occlusion (LVO).^1,5^ Several guidelines have now endorsed tenecteplase as an alternative either for all eligible patients or the LVO subset.^6–8^ The optimal stroke TNK dose is 0.25 mg/kg as there is clinical trial evidence of increased bleeding risk and worse outcomes with 0.4 mg/kg dose.^9^ However, real-world observational data are limited by potential temporal or inter-cluster confounding as before-and after or treatment cluster (e.g. by center or stroke type such as LVO vs non-LVO) comparison are the usual employed methodologies.^2,4,7^ The NZ Central Region Stroke Network was an early universal adopter, but had to revert to alteplase following a global tenecteplase shortage. The resultant three different treatment phases provide a unique opportunity to address potential temporal confounding.

## Methods

We extracted data from a compulsory national stroke reperfusion register comparing all adult patients treated in Central NZ with alteplase from 1 January 2018 to 1 March 2020 (phase 1) to those treated with tenecteplase from 2 March 2020 to 14 February 2021 (phase 2) and those treated with alteplase from 15 February 2021 to 31 December 2022 (phase 3). This register is pragmatic and primarily for assessing the type, efficacy, safety and time metrics of reperfusion therapy and hence comprehensive data on comorbidities and other variables such as risk factors are not routinely collected. The Central Region of NZ comprises 1,166,333 people serviced by one tertiary, four provincial hospitals, and four rural hospitals. Population-based thrombolysis rates were calculated per 100,000 person years. The primary efficacy outcome was 3-month modified Rankin Scale (mRS) (10–16 weeks; doctors/nurses collected; unblinded; 15% retrospective chart-review) using ordinal shift analysis. The main safety outcome was sICH using ECASS 3 criteria. Secondary patient outcomes included mRS dichotomized (0–2vs 3–6), death by day seven, and angioedema. We also assessed four process outcomes: door-to-needle time overall and limited to non-thrombectomy centers (where patients require transfer to another center to undergo thrombectomy), and needle-to-groin time for those who did undergo thrombectomy both overall and by non-thrombectomy center. For the main analysis we compared all alteplase treated patients with tenecteplase treated patients. In addition, we completed two sensitivity analyses: (1) tenecteplase compared to only the second alteplase phase and (2) first alteplase to second alteplase phase.

Data was analyzed using descriptive statistics, Mann-Whitney *U* test for non-parametric, and logistic regression for dichotomous and ordinal variables. Regression models included hospital, age, pre-morbid mRS, baseline National Institute of Health Scale (NIHSS), onset-to-needle time, and thrombectomy. Statistical analysis was performed using Stata IC 17.

NZ Health and Disability Ethics Committee approved registry data use without individual patient consent (HDEC 19STH/55). Authors (AR/AT) could access the complete centrally cleaned registry dataset and the RECORD reporting checklist was used. Study data are available from the corresponding author upon reasonable request.

## Results

A total of 1408 patients were thrombolyzed during the study period: 554 alteplase pre-switch (phase 1), 286 during switch to tenecteplase (phase 2), and 568 once returned to alteplase (phase 3). Population-based thrombolysis rates rose over time (22.0 vs 23.3vs 27.1 per 100,000/year by phase respectively). Pre-hospital onset-to-door times were longer during the tenecteplase phase (phase 2), thrombectomy rates were higher during the tenecteplase and second alteplase phases (phases 2 and 3), and baseline mRS was higher in later phases (mRS 0–2 90.6% phase 1, 85.9% phase 2, and 84.3% phase 3). Additional baseline characteristics are displayed in Table 1.

**Table 1.** Baseline characteristics.

	Alteplase (Phase 1) *N* = 554	Tenecteplase (Phase 2) *N* = 286	Alteplase (Phase 3) *N* = 568	Alteplase (Phase 1+3) *N* = 1121	*p* Tenecteplase vs Alteplase (all)
Age, mean (SD)	71.8 (14.6)	71.7 (14.5)	73.0 (13.9)	71.7 (14.5)	0.47
Sex, female *n* (%)	252 (45.5)	140 (47.2)	257 (45.3)	509 (45.4)	0.60
Ethnicity, *n* (%)^*^					0.27
Europeans	443 (79.8)	240 (83.9)	432 (78.8)	873 (79.3)	
NZ Māori	69 (12.4)	30 (10.5)	77 (14.1)	146 (13.3)	
Pacific	21 (3.78)	11 (3.9)	13 (2.4)	34 (3.0)	
Asian	15 (2.7)	4 (1.4)	18 (3.3)	33 (3.0)	
Other	6 (1.1)	1 (0.4)	8 (1.46)	15 (1.35)	
Pre-stroke mRS, median (IQR)^§^	0 (0–1)	0 (0–1)	0 (0–1)	0 (0–1)	0.029
NIHSS, median (IQR)	8 (5–15)	7 (5–15)	8 (5–15)	8 (5–15)	0.54
Wake-up stroke, *n* (%)*	17 (4.6)	21 (7.4)	29 (5.2)	46 (5.0)	0.12
Onset to door time, median (IQR)	80 (50–118)	89.5 (56–140)	80 (50–128)	80 (50–120)	0.007
Onset to needle time >4.5 h, *n* (%)	17 (3.1)	25 (8.7)	36 (6.3)	53 (4.7)	0.01
Thrombectomy, *n* (%)	34 (6.1)	44 (15.4)	77 (13.6)	111 (9.9)	0.008

SD: standard deviation; IQR: interquartile range. *Ethnicity in phase 3 has 20 (3.5%) missing values. ^§^Pre-mRS spread – Phase 1 (Alteplase): 0 = 73.1%, 1 = 10.5%, 2 = 7.0%, 3 = 7.7%, 4 = 1.7%. Phase 2 (tenecteplase): 0 = 61.5%, 1 = 14.6%, 2 = 9.8%, 3 = 12.00%, 4 = 2.2%; Phase 3 (Alteplase): 0 = 62.7%, 1 = 12.9%, 2 = 9.4%, 3 = 13.1%, 4 = 1.9%; All Alteplase: 0 = 68.0%, 1 = 11.7%, 2 = 8.1%, 3 = 10.4%, 4 = 1.8%. *Missing values phase 1 = 197 (36%), phase 2 = 1 (0.4%) phase 3 = 13 (2.3%).

See Supplemental Tables 1 and 2 for additional baseline variable comparisons.

Compared to alteplase treated patients (phases 1 and 3), tenecteplase patients (phase 2) had a higher adjusted odds ratio (aOR) (95% confidence interval) of favorable outcomes defined as mRS ordinal shift analysis [1.43 (1.11–1.85); *p* = 0.01)] and lower odds of sICH [0.29 (0.09–0.95); *p* = 0.04] (Table 2).

**Table 2.** Patient outcomes tenecteplase (phase 2) versus all alteplase patients (phase 1 and 3).

	Tenecteplase *N* = 286	Alteplase *N* = 1121	OR	aOR Model 1^a^	Adjusted *p*
3-month mRS (shift analysis)			1.14 (0.91–1.44)	1.43 (1.11–1.85)	0.01
3-month mRS (0–2) (%)	176/279^*^ (63.1)	617/1025^§^ (60.2)	1.17 (0.93–1.48)	1.47 (1.01–2.15)	0.04
Death by day 7 (%)	20/286 (7.1)	116/1121 (10.3)	0.63 (0.39–1.04)	0.60 (0.34–1.06)	0.08
sICH (%)	3/286 (1.1)	40 /1121(3.6)	0.29 (0.09–0.93)	0.29 (0.09–0.95)	0.04
Angioedema (%)	1/286 (0.35)	15/1121 (1.34)	0.26 (0.03–1.97)	0.20 (0.03–1.56))	0.12

a Model 1 adjusted for age, pre-mRS, onset-to-needle-time, NIHSS, thrombectomy, hospital; *missing values *n* = 7 (2.5%); ^§^missing values *n* = 83 (7.4%). Additional models displayed in supplement.

Comparing tenecteplase to only the most recent alteplase (phase 3), similar results were noted except the dichotomous 3-month mRS result, which became non-significant (Table 3). There was no difference in patient outcome between the two alteplase phases (Table 4).

**Table 3.** Secondary analyses – Tenecteplase (phase 2) versus most recent Alteplase phase (phase 3).

	Tenecteplase *N* = 286	Alteplase phase 3 *N* = 568	OR	aOR Model 1^a^	Adjusted *p*
3-month mRS (shift analysis)	176/279^*^ (63.1)	287/485^§^ (59.2)	1.27 (0.89–1.64)	1.39 (1.05–1.84)	0.02
3-month mRS (0–2)	176/279^*^ (63.1)	287/485^§^ (59.2)	1.19 (0.87–1.59)	1.23 (0.82–1.85)	0.31
Death by day 7	20/283 (7.1)	52/487 (9.7)	0.70 (0.41–1.20)	0.67 (0.37–1.23)	0.19
sICH	3/286 (1.1)	21/567 (3.7)	0.28 (0.082–0.93)	0.26 (0.076–0.88)	0.03
Angioedema	1/286 (0.35)	8/568 (1/41)	0.25 (0.03–1.97)	0.27 (0.03–2.29)	0.23

§ Missing values: 83 (14.6%); *Missing values 7 (2.5%).

**Table 4.** Secondary analysis Alteplase (phase 1) versus Alteplase (phase 3).

	Alteplase phase 3 *N* = 568	Alteplase phase 1 *N* = 554	OR	aOR Model 1^a^	Adjusted *p*
3-month mRS (shift analysis)	287/485^§^ (59.2)	330/540^*^ (61.1)	0.93 (0.84–1.04)	1.04 (0.93–1.17)	0.51
3-month mRS (0–2)	287/485^§^ (59.2)	330/540^*^ (61.1)	0.96 (0.85–1.09)	1.03 (0.91–1.15)	0.63
Death by day 7	52/487 (9.7)	64/553 (11.6)	0.91 (0.74–1.10)	0.91 (0.72–1.14)	0.41
sICH	21/567 (3.7)	19/553 (3.4)	1.04 (0.76–1.43)	1.09 (0.78–1.53)	0.61
Angioedema	8/568 (1/41)	7/553 (1.3)	1.06 (0.63–1.75)	0.89 (0.50–1.57)	0.69

* Missing values 14 (2.5%). ^§^Missing values: 83 (14.6%).

**Figure 1.**
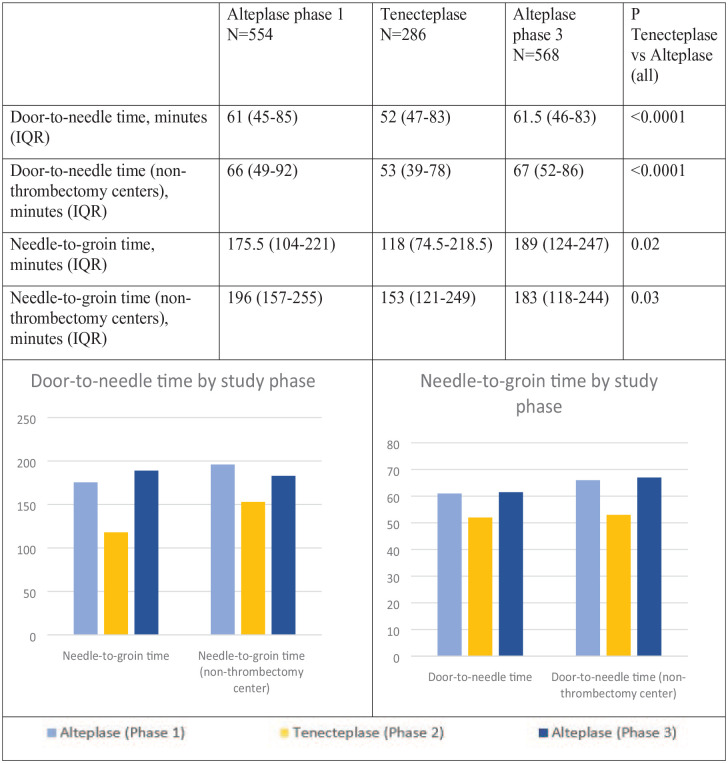
Door-to-Needle and Groin times by phase; overall and for non-thrombectomy centers.

Door-to-needle -time during the tenecteplase phase was faster compared with alteplase [52 (47–83) and 61 (45–84) minutes respectively]. Needle-to-groin time was also shorter during the tenecteplase phase compared with alteplase overall [118 (74.5–218.5) to (185 (1118–255), respectively]. Similar patterns were observed when limited to patients presenting to non-thrombectomy centers. Times by individual phase are displayed in Figure 1.

## Discussion

In this opportunity real world study of unselected consecutively thrombolysed patients at tertiary, urban and non-urban secondary, and small rural hospitals, we found no evidence of harm related to tenecteplase use compared with alteplase and overall improved patient outcomes, with fewer adverse events, and reduced treatment delays. We previously presented similar findings looking at only the first two phases,^5^ however, potential other intervening temporal confounders presented a limitation especially given the significant rise in thrombectomy rates between phases 1 and 2. This new data arising from the subsequent forced reversion to alteplase provides significant reassurance regarding the validity of the original results. In addition, the overall increased sample size has resulted in greater study power around the reduction of adverse events.

One finding that differs between our and other observational and trial data is the association of tenecteplase with reduced treatment delays. In the real-world setting, the 1-h alteplase infusion can present a barrier to in-CT thrombolysis due to concerns around a long gap between bolus and infusion, especially where tenecteplase boluses are routinely given before advanced imaging, and barriers to helicopter transport with an infusion pump. The demonstrated reduction in time delays with tenecteplase is even more significant consdiering that the tenecteplase phase coincided with the peak health system impact of the covid pandemic in New Zealand. It is possible that this real-world benefit in treatment speed contributes to reduced adverse and overall better patient outcomes with tenecteplase. Our sICH rates in tenecteplase and Alteplase groups aligns with other published data although higher sICH rates have been reported in some tenecteplase trials.^2,3,9^ This difference may be attributable to higher doses, including use of a weight-tiered dosing schemes in relevant trials.^3,9^

The ability to compare with alteplase cases both pre- and post-tenecteplase implementation is a unique feature of this study that addresses temporal confounding. The complete case ascertainment with inclusion of all consecutively treated patients at all participating hospitals is a key strength of this study that eliminates selection bias and provides real-world applicability. Another key strength is the near complete 3-month follow-up data acquisition allowing conclusions to be drawn about associated benefit in 3-month functional outcome; this was a limitation of the recent publication from our multi-national CERTAIN collaborative which focussed on safety outcomes.^4^

This study has several limitations. This is a registry based, unblinded data set with associated risk for bias and confounding by unmeasured/unknown factors. Secondly, it is unclear whether the intervening COVID-19 pandemic may have had an effect and this was not adjusted for in this study. The impact of COVID-19 on NZ was quite different from other countries as evidenced by the fact that there was not a single COVID-19 positive stroke patient in our data set. Processes of care were mainly impacted during March-May 2020 when new national isolation procedures were being implemented. This period occurred during the tenecteplase phase and thus negatively impacted that phase only. While a multi-center study, it is New Zealand based, which may limit generalizability especially to population of predominantly non-European decent. The inclusion of multiple non-tertiary and even rural hospitals allows generalizability beyond the tertiary setting.

Our results provide high-quality real-world confirmatory evidence for safety and pragmatic advantages of tenecteplase when used in acute ischemic stroke.

## Supplemental Material

sj-docx-1-eso-10.1177_23969873231187436 – Supplemental material for Tenecteplase real-world data: A three phase sequential comparison Click here for additional data file. Supplemental material, sj-docx-1-eso-10.1177_23969873231187436 for Tenecteplase real-world data: A three phase sequential comparison by Anna Ranta, Alicia Tyson, Bhavesh Lallu, Teddy Y Wu, Martin Punter, Csilla Manoczki, John Chalissery, Akesh Pillai, Karim Mahawish, Roldan Conde, Marianne Falconer, Karyn Wills, Chaminda Gunawardana, Suzanne Busch and John Gommans in European Stroke Journal

sj-docx-2-eso-10.1177_23969873231187436 – Supplemental material for Tenecteplase real-world data: A three phase sequential comparison Click here for additional data file. Supplemental material, sj-docx-2-eso-10.1177_23969873231187436 for Tenecteplase real-world data: A three phase sequential comparison by Anna Ranta, Alicia Tyson, Bhavesh Lallu, Teddy Y Wu, Martin Punter, Csilla Manoczki, John Chalissery, Akesh Pillai, Karim Mahawish, Roldan Conde, Marianne Falconer, Karyn Wills, Chaminda Gunawardana, Suzanne Busch and John Gommans in European Stroke Journal
