# Supplementary material for: Tenecteplase real-world data: A three phase sequential comparison
Source: Eur Stroke J. 2023 Jul 25;8(4):942–6. doi: 10.1177/23969873231187436 (PMC10683726; doi:10.1177/23969873231187436)
Supplement: sj-docx-2-eso-10.1177_23969873231187436 – Supplemental material for Tenecteplase real-world data: A three phase sequential comparison [file sj-docx-2-eso-10.1177_23969873231187436.docx]

Supplementary Data

Supplementary Table 1: Baseline characteristics additional comparisons for individual study phases

|  | Alteplase (Phase 1)  N=554 | Tenecteplase  (Phase 2)  N=286 | Alteplase  (Phase 3)  N=568 | Alteplase  (Phase 1+3)  N=1121 | P  Phase  2 vs 3 | P  Phase  1 vs3 |
| --- | --- | --- | --- | --- | --- | --- |
| Age, mean (SD) | 71.8 (14.6) | 71.7 (14.5) | 73.0 (13.9) | 71.7 (14.5) | 0.21 | 0.15 |
| Sex, Female n(%) | 252 (45.5) | 139 (48.6) | 257 (45.3) | 509 (45.4) | 0.35 | 0.91 |
| Ethnicity, n(%)* |  |  |  |  | 0.13 | 0.08 |
| Europeans | 441 (79.8) | 237(83.8) | 432 (78.8) | 873 (79.3) |  |  |
| NZ Māori | 69 (12.5) | 30 (10.6) | 77 (14.1) | 146 (13.3) |  |  |
| Pacific | 21 (3. 8) | 11 (3.9) | 13 (2.4) | 34 (3.0) |  |  |
| Asian | 15 (2.7) | 4 (1.4) | 18 (3.3) | 33 (3.0) |  |  |
| Other | 6 (1.1) | 1 (0.4) | 8 (1.46) | 15 (1.35) |  |  |
| Pre-stroke mRS, median (IQR)^§^ | 0 (0-1) | 0 (0-1) | 0 (0-1) | 0 (0-1) | 0.77 | <0.001 |
| NIHSS, median (IQR) | 8 (5-15) | 7 (5-15) | 8 (5-15) | 8 (5-15) | 0.94 | 0.94 |
| Wake-up stroke, n(%)* | 17 (4.6) | 21 (7.4) | 29 (5.2) | 46 (5.0) | 0.22 | 0.64 |
| Onset to needle time >4.5 hrs, n(%) | 17 (3.1) | 25 (8.7) | 36 (6.3) | 53 (4.7) | 0.2 | 0.01 |
| Onset to groin time >6 hrs, n(%) | 11/34 (32.4) | 14/44 (31.8) | 42/77 (54.6) | 53/111 (47.8) |  |  |
| Onset to door time, median (IQR) | 80 (50-118) | 89.5 (56-140) | 80 (50-128) | 80 (50-120) | 0.069 | 0.50 |
| Thrombectomy, n(%) | 34 (6.1) | 44 (15.4) | 77 (13.6) | 111 (9.9) | 0.47 | <0.001 |

^*^Ethnicity data for phase 3 has 20 missing values. ^§^Pre-mRS spread – Phase 1 (Alteplase): 0=73.1%, 1=10.5%, 2=7.0%, 3=7.7%, 4=1.7%. Phase 2 (Tenecteplase): 0=61.5%, 1=14.6%, 2=10.2%, 3=12.00%, 4=2.2%; Phase 3 (Alteplase): 0=62.7, 1=12.9%, 2=9.4%, 3=13.1%, 4=1.9%; All Alteplase: 0=68.0%, 1=11.7%, 2=8.1%, 3=10.4%, 4=1.8%.

*Missing values phase 1 =197 (36%), phase 2= 1(0.4%) phase 3=13 (2.3%)

Supplementary Table 2: Additional baseline characteristics (optional reporting in the register) – due to high proportion of missing values not included in main manuscript

|  | Alteplase (Phase 1)  N=553 | Tenecteplase  (Phase 2)  N=286 | Alteplase  (Phase 3)  N=567 | Alteplase  (Phase 1+3)  N=870 | P  TNK vs tPA (all) | P Phase 2 vs phase 3 | P  Phase 1 vs phase 3 |
| --- | --- | --- | --- | --- | --- | --- | --- |
| Hypertension, n/n(%) | 128/211 (60.7) | 159/209 (76.1) | 202/319 (63.2) | 330/530 (62.3) | <0.001 | 0.002 | 0.54 |
| Diabetes, n/n(%) | 36/211 (17.1) | 41/209 (19.5) | 56/319 (17.6) | 330/530 (62.2) | <0.001 | 0.55 | 0.54 |
| Dyslipidemia, n/n(%) | 74/211 (36.6) | 81/209 (38.8) | 73/319 (22.9) | 148/530 (27.9) | 0.004 | <0.001 | 0.001 |
| Atrial Fibrillation, n/n(%) | 89/211 (45.2) | 81/209 (38.8) | 130/319 (40.8) | 219/530 (41.3) | 0.52 | 0.65 | 0.74 |
| Current smoker, n/n(%) | 36/211 (17.1) | 23/209  (11) | 26 (8.2) | 62/530 (11.7) | 0.79 | 0.27 | 0.002 |
| Prior stroke/TIA, n/n(%) | 81/211 (28.4) | 68 (32.5) | 90/319 (28.2) | 171530 (32.3) | 0.94 | 0.29 | 0.01 |
| Blood glucose on admission, median mean, median (IQR)* | 6.4 (5.6-7.9)  7.4 (3.2) | 6.4 (5.6-7.9)  7.2 (2.5) | 6.3 (5.5-8.3)  7.1 (2.8) | 6.4 (5.6-7.8) | 0.58 | 0.38 | 0.34 |
| Systolic blood pressure on admission, median (IQR)** | 155 (140-172)  157.5 (25.8) | 154 (140-171)  155.5 (22.4) | 150 (137-166)  151.4 (22.8) | 151 (139-169)  153.9 (24.3) | 0.25 | 0.02 | 0.001 |

*Missing values: Phase 1 219 (39.6%), Phase 2=59 (20%) Phase 3=150 (26%) **Missing values Phase 1=183 (33%), Phase 2=26 (9%), Phase 3=60 (10%)

Supplementary Table 3: Additional models adjusting for baseline blood glucose (model 2) and systolic blood pressure (model 3) – excluded from main analysis as model fit not improved, results similar to main model except wider confidence intervals due to reduction in sample size given high number of missing values for these variables (Blood Glucose 30.6% and Blood Pressure 19%)

|  | Tenecteplase  N=286 | Alteplase  N=1121 | aOR Model 2^*^ | aOR Model 3^**^ |
| --- | --- | --- | --- | --- |
| 3-month mRS (shift analysis) |  |  | 1.35 (1.04-1.80) | 1.4 (1.02- 1.74) |
| 3-month mRS (0-2), n/n(%) | 176/279 (63.1) | 617/1025 (60.6) | 1.34 (0.87-2.05) | 1.25 (0.84-1.84) |
| Death by day 7, n/n(%) | 20/283 (7.1) | 116 (10.6) | 0.60 (0.31-1.17) | 0.72 (0.40-1.31) |
| sICH, n/n(%) | 3/286 (1.1) | 40 (3.6) | 0.34 (0.10-1.15) | 0.27 (0.08-0.89) |
| Angioedema, n/n(%) | 1/286 (0.35) | 15/1121 (1.34) | 0.23 (0.03-1.87) | 0.25 (0.03-2.07) |

^*^ Same as main model but baseline blood glucose added; ^**^Same as main model but systolic blood pressure on presentation added
